# Racial Disparities in Outpatient Substance Use Disorder Treatment Completion: Trends and Changes from 2004 to 2024

**DOI:** 10.3390/ijerph22020278

**Published:** 2025-02-14

**Authors:** Monica F. Wright

**Affiliations:** Northwell, New Hyde Park, NY 11004, USA; mwright34@northwell.edu

**Keywords:** addiction treatment, treatment completion, substance use treatment, completion rates, racial disparities

## Abstract

Racial disparities have been found in outpatient substance use disorder (SUD) treatment completion rates. Improvements in access to treatment have sought to decrease these disparities and increase treatment engagement and success. To understand outcomes among different racial groups across time, we assessed (1) completion rates, (2) racial representation among patients who have completed treatment, (3) general representation of racial groups within treatment, and (4) treatment length between 2004 and 2024. “Completion” is defined as “meeting all treatment goals”. Chi-squared analyses suggest significant differences among racial groups within the completed ($x^{2}\left(15\right)=158.0,\;p=<0.001)$, not-completed ($x^{2}\left(15\right)=561.75,\;p=<0.001)$, and other ($x^{2}\left(15\right)=186.19,\;p=<0.001)$ groups across time. Asian and Other/Multiracial patients experienced the greatest improvement in both representation within treatment overall and proportional representation within the “completer” group over time, despite their overall completion rates fluctuating. White and Black/African American patients decreased in representation, completion rates, and representation in the “completer” group over time, with a peak in 2004–2009. In terms of length of stay, White patients remained in treatment the longest across time, *F*(5, 4198) = 24.605, *p* < 0.011, and treatment length increased for other racial groups. While disparities in completion rates decreased and racial representation in treatment increased, discrepancies persist. We discuss these findings within the context of evolving patient populations and changes in treatment provision (e.g., harm reduction frameworks).

## 1. Introduction

Substance misuse is among the most critical public health concerns of our time [1]. Substance use disorders (SUDs) and their commonly co-occurring mental health disorders (e.g., major depressive disorder and anxiety disorders) represent the leading cause of disability globally [2,3,4]. While SUDs exist across racial groups within the United States, American Indian/Alaska Native populations have consistently suffered from the highest prevalence rates (12.2% in the past year [5]) compared to other racial groups [6,7]. Individuals who identify with multiple racial groups have the second highest prevalence rates (past year 10.8% [5]), followed by Whites (8.2%), Blacks/African Americans (7.6%), Hawaiians/Pacific Islanders (7.3%), and Asians (4.3%) [5]. Among ethnic groups, Hispanic individuals often have rates higher than other ethnic groups, with a past-year prevalence of 7.5% [5].

Although prevalence rates illuminate how widespread SUDs are in certain racial groups, they do not necessarily capture the functional impairment associated with SUDs in each population. For example, Black/African American individuals have lower SUD rates compared to other racial groups; however, the pernicious effects of systemic racism [8,9,10] have led them to experience a higher burden of overdose deaths, physical disease secondary to substance use, criminal justice involvement due to drug-related crimes, and comorbid psychiatric disorders [8,11,12]. Racial groups with both high SUD prevalence and higher functional impairment should be prioritized in specialized SUD treatment and would ideally seek out and receive treatment at higher rates than other racial groups.

### 1.1. Race/Ethnicity and SUD Treatment Initiation

Regrettably, data have historically suggested that outpatient SUD treatment is utilized by White patients more than all other racial groups [13], with few exceptions [14]. Black/African American individuals are repeatedly found to be the least likely to initiate professional treatment compared to any other race or ethnicity, especially if the service exclusively focuses on substance use treatment, compared to integrated substance use and mental health care services [13,15]. Studies investigating reasons for this discrepancy have highlighted access, insurance coverage, and mistrust of medical systems as major barriers [16,17].

Multiple initiatives have emerged to help address the discrepancy in treatment access and utilization around the world. The World Health Organization recommends public health approaches to substance use which emphasize harm reduction frameworks [18], defined as policies, programs, and practices that minimize the negative health, social, and legal impacts associated with substance use [19]. Such approaches have had far-reaching impacts, from clean needle and drug treatment programs in African countries struggling with infectious diseases (e.g., Human Immunodeficiency Virus) [19,20] to the decriminalization of substances in countries such as Canada and Portugal [18]. In the United States, public health approaches have led to initiatives such as Screening, Brief Intervention, and Referral to Treatment (SBIRT) within medical and community organizations to help identify and connect individuals with substance use treatment [21].

These public health and harm reduction initiatives have contributed to important policy changes. For example, the Affordable Care Act of 2010 in the United States promoted widespread insurance coverage for substance use disorders and led to a significant increase in Black/African American patients seeking out SUD treatment [16,17]. In the years following this act, White and Black/African American patients became equally likely to utilize specialty substance use services [22].

The increased focus on and support for individuals with SUDs has also led to better quality services. Treatment programs are increasingly incorporating mental health treatment and moving away from traditional “abstinence-only” models, which is inviting and attracting a more diverse patient population; in terms of both demographic diversity and substance use goals [23,24,25]. Advances in Medication Assisted Treatment (e.g., Buprenorphine and Naltrexone) assist patients in managing their use and encourage patients to remain in treatment for longer periods of time [26,27].

These major changes in the approach to substance use treatment have contributed to a more diverse patient population, and fewer racial discrepancies in treatment initiation [15,22,28]. They have also contributed to a shift in treatment provision, connection to treatment, and treatment availability/accessibility. As patient populations increase and more resources are dedicated to substance use treatment, there is an assumption that patients will have better outcomes over time, despite little research assessing completion rates over time.

### 1.2. Race and SUD Treatment Completion

Definitions of treatment completion have taken many forms in the literature. Historically, treatment completion was defined by the achievement of abstinence, and criteria for entering treatment were extremely exclusive and abstinence-focused [29]. More recently, treatment programs are using treatment length or the nature of the discharge (e.g., planned versus unexpected discharge) [30] to evaluate completion. Recent data using these metrics consistently find completion rates to be lower among Black/African American compared to White patients [17,31,32,33] and Asians/Pacific Islanders to have higher odds of completing treatment [30,34]. Data on treatment length and number of sessions are useful metrics for engagement; however, these metrics do not indicate whether engagement has contributed to improved clinical outcomes.

The current project aims to address these concerns and defines treatment completion as “meeting all treatment goals”. This project specifically aims to understand whether rates of treatment completion have improved over time, as would be assumed given the major policy changes to facilitate such improvements, and whether discrepancies in treatment representation have decreased. This project also aims to better understand whether racial differences in treatment length and time to treatment completion persist.

## 2. Materials and Methods

### 2.1. Data Source

This project assessed discharge data among patients from five outpatient substance use clinics in New York (NY) State from January 2004 to November 2024 as part of a quality improvement initiative to improve patient care. All 5 SUD clinics affiliated with a private hospital system in New York were included in the analyses. Discharge data were extracted from patient records and included admission date, discharge date, reason for discharge, racial identity, sex, date of birth, and psychiatric diagnoses (including substance use disorder diagnosis).

Patients were separated into 4 time points: Group 1: 2004–2009, Group 2: 2010–2014, Group 3: 2015–2019, and Group 4: 2020–2024. The first group spans 6 years instead of 5, to allow for more equal group sizes, as the patient census was lower in these years.

### 2.2. Defining Treatment Completion

The discharge data were assessed to determine the reason for discharge. This project focused on the discharge status “completed”, which was defined as “having completed all treatment goals in treatment plan”. While this definition of “completed” has been consistent since 2004, it is useful to understand the context in which patient progress was evaluated. In the early 2000′s, most programs in NY State focused exclusively on abstinence and would discharge patients if they did not remain abstinent during treatment [35]. Between 2004 and 2009, the outpatient programs included in this quality improvement initiative were abstinence-focused and had strict rules around treatment adherence. The only treatment goal being tracked was abstinence. Programs rarely accepted patients who were interested in focusing on moderation or who wanted to explore other goals within treatment, such as mental health goals. These programs also rarely retained patients who engaged in ongoing substance use or who experienced relapse. In the 2010s, there was a clear shift towards harm reduction and more acceptance of individuals entering treatment with a range of substance use goals. Over the last decade, the outpatient programs included in this project have formally identified themselves as harm reduction and dual-diagnosis and admit patients who have a wide range of treatment and mental health goals [28].

### 2.3. Analytic Approach

Completion rates for this project were calculated by dividing the number of completers by the number of attempters, that is, completers/completers + non-completers. Individuals who were discharged due to the requirement of medical care or a different level of care, death, or relocation were not considered non-completers but rather to have left treatment due to practical or non-clinical reasons. These individuals were not included in the completion rate analyses.

Using these criteria, overall completion rates were calculated, and then completion rates in each racial group across time were calculated. Chi-squared tests were utilized to understand whether there were differences in the number of patients in each racial group who completed, did not complete, or left for other reasons. To assess whether patients were completing treatment at similar rates, time to treatment completion was assessed using a univariate analysis of variance, where length of stay until successful discharge among different racial groups was compared across time. Due to the sensitive nature of these patient data, raw data that are de-identified are only available upon request from the corresponding author. This study was reviewed by the Institutional Research Board (IRB) within our hospital system and deemed quality improvement rather than human subject research. Therefore, it was exempt from requiring IRB approval; HSRD23-0055.

## 3. Results

### 3.1. Demographics

Discharge data from 20,228 patients in the Addiction Services Program of a private hospital in NY were available. Of these patients, 3226 (15.9%) did not complete their intake process and were screened out before admission. Reasons for screening patients out included not meeting criteria for an SUD diagnosis, requiring a different level of care, or not completing the intake process. Therefore, 17,002 patients were admitted into substance use treatment between 2004 and 2024.

Most patients were biologically male (64.4%) and White (63.1%), followed by Black/African American (21.7%), Other/Multiracial (11.1%), Asian (2.4%), and Native American/Alaska Native (0.3%). The majority struggled with an alcohol use disorder (AUD; 37.1%), followed by opiate use disorder (OUD; 24.7%), cannabis use disorder (18.0%), cocaine use disorder (8.7%), and benzodiazepine use disorder (1.8%). Polysubstance use, sedative use disorder, hallucinogen use disorder, and other substance use disorders together comprised 9.5% of the sample. Approximately one-third (27.6%) had a psychiatric comorbidity in their chart. Length of stay among discharged patients ranged from 30 days to 9624 days (almost 26 years). The average length of stay was approximately one year (*M* = 367.33, *SD* = 711.74).

### 3.2. Discharge Reason

Discharge status was documented for each patient upon discharge, and reasons for discharge are outlined in Table 1. Over half (51.4%) of admitted patients were discharged prior to treatment completion, 24.6% completed all treatment goals, and 24.0% were discharged for other reasons (e.g., patient death).

### 3.3. Representation of Racial Groups in Treatment Across Time

Table 2 outlines the discharge statuses by racial group over time, while also presenting the proportion of each racial group in the patient census in that year grouping. Chi-squared analyses indicate that, over time, there were significant differences among racial groups within the completed ($x^{2}\left(15\right)=158.0,\;p=<0.001)$, not-completed ($x^{2}\left(15\right)=561.75,\;p=<0.001)$, and other ($x^{2}\left(15\right)=186.19,\;p=<0.001)$ groups across time. Across discharge status, there were also significant differences within racial groups across time $x^{2}\left(15\right)=823.24,\;p=<0.001.$ Differences across years within the races are denoted by subscript letters in Table 2. Numbers with the same subscript letters are not statistically different from each other, and numbers with different subscript letters are statistically different at *p* < 0.05.

Specifically, over time, the proportion of White patients in treatment decreased significantly over time, peaked between 2015 and 2019 (64.7% of the patient population within those years), and then decreased in 2020–2024. The overall representation of Black/African American patients increased, with a peak between 2010 and 2014 (26.3% of patients within those years), and then decreased. The population of Asian patients fluctuated over time, increasing overall, with a peak between 2020 and 2024 (5.6% of patients in those years). The American Indian/Alaska Native population also peaked between 2015 and 2019 (0.4% of patients within that year grouping). Other/Multiracial patients increased steadily in representation and peaked between 2020 and 2024 (20.5% of patients within that year grouping). Individuals with unknown race were most prevalent between 2010 and 2014 (0.7% of patients within that year grouping).

### 3.4. Completion Rates

Completion rates, overall, are highest among Native American/Alaska Native patients. They also fluctuated the most among this population, peaking at 83.33% between 2010 and 2013, likely due to the extremely low numbers of individuals within this racial category over time causing wide variability year to year. Individuals with no race documented on file had the next highest completion rate, peaking at 100% between 2020 and 2024; again, this high fluctuation is likely due to the very small number of patients who do not have race documented on file. This result is likely due to the improved documentation of race over time and not a result of improved care among individuals with no documented race. Asian patients followed, with rates peaking at 54.10% between 2004 and 2009. White patients had completion rates just below those of Asian patients, with the highest completion rates between 2004 and 2009 (38.44%). Completion rates among White patients decreased slightly over time. Other/Multiracial patients’ completion rates also decreased, with the highest rates between 2004 and 2009 (38.44%). Black/African American patients had the lowest completion rates, which peaked between 2015 and 2019 at 28.4%.

### 3.5. Time to Treatment Completion

A univariate ANOVA was conducted among only those who completed treatment, with length of stay as the dependent variable and race and year grouping as the independent variables. Overall, there were significant differences between racial groups across time and length of stay (*F*(5, 4198) = 24.605, *p* < 0.011) and a significant interaction between racial group and year grouping (*F*(14, 4198) = 1,161,919.35, *p* = 0.003). There were no significant differences over time across racial groups (*p* = 0.10). Across time, however, White patients remained in treatment for significantly longer (*M* = 615.95 days, *SE* = 13.59), almost twice as long, as Asian (*M* = 327.05, *SE* = 52.63, *p* < 0.001), Black (*M* = 362.70, *SE* = 27.15, *p* < 0.001), and Other/Multiracial (*M* =350.04, *SE* = 33.74, *p* < 0.001) patients. White patients did not significantly differ from Native American/Alaska Native (*M* = 345.94, *SE* = 165.54, *p* = 0.10) patients in length of stay.

Across racial groups, the length of time to completion fluctuated significantly (*F*(5, 4198) = 24.69, *p* < 0.001). Specifically, patients remained in treatment for significantly longer between 2020 and 2024 (*M* = 544.74 days, *SE* = 75.52, *p* = 0.01), compared to 2010–2014 (*M* = 293.63). Within racial groups, length of stay until completion increased among Asian, Black/African American, Other/Multiracial, and White patients, whereas Native American/Alaska Native patients completed treatment in less time than before. See Table 3.

## 4. Discussion

The landscape of substance use treatment has dramatically changed over the last two decades. Until recently, the abstinence-only model reigned, and few resources were allotted to substance use treatment programs [36]. In the early 2000s, programs were primarily accessible and available to individuals with relatively higher means, who had government assistance (e.g., Medicaid), or who were mandated legally to attend [37]. In this sample, the racial make-up of the first time span (2004–2009) was primarily White and African American/Black patients, making up 93.1% of the total treatment sample in those years. The former remained in treatment for longer and had almost double the rate of completion, compared to the latter. This was also the time point where both White and African American/Black patients represented a statistically higher proportion with the “completers” group than any other time frame. Individuals from other racial groups were highly under-represented, especially within the “completers” group, see Table 2. This may have a been a time where African American/Black patients were experiencing the most external pressure to complete treatment and may have been mandated to remain abstinent—a consequence of the war on drugs. Thus, completion rates in this time may better reflect coercion than success.

As policies around treatment access, criminal justice reform, public health approaches, and harm reduction increased, the diversity in patient populations also increased, and individuals identifying as Black/African American began completing treatment at higher rates overall (despite decreasing their representation in the “completers group”) and remaining in treatment for longer. Other racial groups, such as Asian Americans became more highly represented, and their completion rates increased. By 2020, New York experienced significant upheaval with the arrival of COVID-19 and the Black Lives Matter movement [38]. Fewer Black/African American individuals entered treatment around this time [28]. Asian patients, on the other hand, appeared motivated to enter treatment when Telehealth became an option, and may have needed more support as anti-Asian sentiment increased during the COVID-19 pandemic. The years 2020–2024 saw a surge of representation among Asian patients.

Other research analyzing data from the same hospital system found that the transition to Telehealth treatment around this time influenced the demographic organization of substance use programs significantly, and specifically decreased the population of Black/African American patients [28], possibly accounting for the drop in completion rates around this time. It is important to consider that, while representation and completion rates have fluctuated over time among different racial groups, the disparities in completion rates between racial groups is narrowing. Between 2004 and 2009, completion ranged from 21 to 83% among racial group. By 2020–2024, the groups ranged only 12 percentage points (from 20 to 32%). The reduction in significant disparities between groups is likely partly accounted for by the higher representation of each group, and a “regression towards the mean” phenomenon. For example, completion rates naturally fluctuate more significantly when some groups are under-represented in the overall sample. For example, in 2004–2009, the Other/Multiracial group included 173 patients and had a completion rate of 45.5%. Their representation in the “completer” group also statistically significantly increased at each time frame. In 2020–2024, the representation within this group increased to 746 patients and the completion rate dropped to 26.2%. It is possible that this drop indicates that substance use treatment became less effective over time for this population; however, it is more likely that a more diverse patient population, with more diverse goals, needs and presentations entered treatment and experienced more varying outcomes as a result and that the more recent completion rates have fewer statistical errors due to larger sample sizes in general. To illustrate how treatment is becoming more representative over time, Table 4 shows general demographics within New York State (census.gov), compared to demographics within the present sample.

Patients who continue to be under-represented, and compared to their community representation, are Black/African American and Asian patients. However, it is important to recall that prevalence rates of SUDs in these groups are lower overall in the community, compared to White patients. The representation of patients who identify as American Indian/Alaska Native is far below what would be expected given the prevalence rates of SUDs in this population. This may be a population that requires more outreach and support to access treatment. Additionally, although representation among Black/African American patients appears to be somewhat consistent with community representation, these patients continue to have poorer outcomes. They are the least represented in the “completer” group and have the lowest completion rates. It is imperative that future research identifies ways to buffer against the ongoing racial disparities in treatment completion rates and continue improving the efficiency and quality of outpatient substance use treatment for this population. It is also important to understand why White patients are remaining in treatment the longest and to assess whether this result is due to difficulty achieving positive outcomes or a preference for remaining in treatment.

Several limitations in this project are important to identify. Due to the observational nature of the project, there was no ability to standardize or control the treatment provision within each program. Naturally, programs evolve greatly over time. It is challenging to directly compare completion rates over time when the programs themselves adopt new ideologies (e.g., harm reduction) and new methods of providing treatment (e.g., more evidence-based care and medication-assisted treatment). Further, completion rates are somewhat tied to the sample size in each population and may be difficult to interpret out of context when certain racial groups are growing exponentially in size.

## 5. Conclusions

The major changes seen in insurance coverage, quality of care, access, and convenience of treatment have led to a more diverse patient population entering and remaining in outpatient substance use treatment in New York. The representation of non-White patients has improved steadily over time (and has more than quadrupled for patients identifying as Other/Multiracial), especially with the advent of Telehealth. The representation of non-White patients is becoming more consistent with community representation. Completion rates, across time, are highest among American Indian/Alaska Native patients, although the sample size remains very small and continues to be far lower than community representation. Asian and White patients have the second highest completion rates, followed by Other/Multiracial and Black/African American patients. While completion rates have not improved over time, there is clear evidence that treatment is becoming more effective for a wider range of patients and is attracting a more complex and diverse population. Further, the length of time to treatment completion is increasing across all groups, aside from Native American/Alaska Native patients, who are completing treatment in less time over the years. White patients take significantly longer to complete treatment compared to most other racial groups. It is important to consider how harm reduction frameworks have partly shaped these shifts over time. It is also important to appreciate that White patients continue to utilize most treatment resources.

Future research is needed to understand whether certain groups require longer courses of treatment to achieve the same gains or whether White patients are preferring to stay in treatment longer and are using outpatient programming as long-term support. Future research is also needed to understand why the representation of Black/African American patients is decreasing and why completion rates are low. This question must be considered within the context of systemic racism and the historical under-utilization of specialized services within this population [8].

## Figures and Tables

**Table 1:** Reasons for discharge.

Documented Reason for Discharge	Frequency (*n*)	Percent
Completed Treatment		
Completed	4190	24.6%
Total Completers	4190	24.6%
Did Not Complete Treatment		
Lost to Contact	4992	29.4%
Noncompliance	241	1.4%
Other	813	4.8%
Unknown	217	1.3%
Withdrew	2468	14.5%
Total Non-Completers	8731	51.4%
Other		
Different Level of Care	1647	9.7%
Died	203	1.2%
Incarcerated	296	1.7%
Medical	131	0.8%
Relocated	1804	10.6%
Total Other	4081	24.0%
Total	17,002	100.0%

**Table 2:** Discharge status by racial group across time.

	Year Grouping					
Discharge Status	Race	2004–2009	2010–2014	2015–2019	2020–2024	Total
Completers						
	White					
	n	791 _a_	726 _b_	703 _b_	501 _b_	2721
	% of total	18.9%	17.3%	16.8%	12.0%	64.9%
	Black/African American					
	n	184 _a_	260 _b_	211 _a_	98 _c_	753
	% of total	4.4%	6.2%	5.0%	2.3%	18.0%
	Asian					
	n	33 _a_	45 _a,b_	59 _b_	45 _b_	182
	% of total	0.8%	1.1%	1.4%	1.1%	4.3%
	Other/Multiracial					
	n	67 _a_	99 _b_	169 _c_	158 _d_	493
	% of total	1.6%	2.4%	4.0%	3.8%	11.8%
	Native American/Alaska Native					
	n	3 _a_	5 _a_	8 _a_	4 _a_	20
	% of total	0.1%	0.1%	0.2%	0.1%	0.5%
	Unknown					
	n	7 _a,b_	12 _b_	2 _a,c_	0 _c_	21
	% of total	0.2%	0.3%	0.0%	0.0%	0.5%
	Total					
	n	1085	1147	1152	806	4190
	% of total	25.9%	27.4%	27.5%	19.2%	100.0%
Non-Completers						
	White					
	n	1267 _a_	1272 _b_	1577 _a_	1030 _c_	5146
	% of total	14.5%	14.6%	18.1%	11.8%	58.9%
	Black/African American					
	n	656 _a_	695 _a_	532 _b_	381 _b_	2264
	% of total	7.5%	8.0%	6.1%	4.4%	25.9%
	Asian					
	n	28 _a_	65 _b_	60 _b_	106 _c_	259
	% of total	0.3%	0.7%	0.7%	1.2%	3.0%
	Other/Multiracial					
	n	80 _a_	176 _b_	319 _c_	445 _d_	1020
	% of total	0.9%	2.0%	3.7%	5.1%	11.7%
	Native American/Alaska Native					
	n	4 _a,b_	1 _b_	7 _a_	8 _a_	20
	%	0.0%	0.0%	0.1%	0.1%	0.2%
	Unknown					
	n	6 _a_	15 _a_	1 _b_	0 _b_	22
	% of total	0.1%	0.2%	0.0%	0.0%	0.3%
	Total					
	n	1085	1147	1152	806	4190
	% of total	25.9%	27.4%	27.5%	19.2%	100.0%
Other						
	White					
	n	646 _a_	767 _a,b_	865 _a,b_	591 _b_	2869
	% of total	15.8%	18.8%	21.2%	14.5%	70.3%
	Black/African American					
	n	197 _a_	220 _a_	176 _b_	81 _c_	674
	% of total	4.8%	5.4%	4.3%	2.0%	16.5%
	Asian					
	n	19 _a_	32 _a_	39 _a_	53 _b_	143
	% of total	0.5%	0.8%	1.0%	1.3%	3.5%
	Other/Multiracial					
	n	26 _a_	76 _b_	133 _c_	143 _d_	378
	% of total	0.6%	1.9%	3.3%	3.5%	9.3%
	American Indian/Alaska Native					
	n	3 _a_	2 _a_	3 _a_	2 _a_	10
	% of total	0.1%	0.0%	0.1%	0.0%	0.2%
	Unknown					
	n	2 _a,b_	5 _b_	0 _a_	0 _a_	7
	% of total	0.0%	0.1%	0.0%	0.0%	0.2%
	Total					
	n	893	1102	1216	870	4081
	% of total	21.9%	27.0%	29.8%	21.3%	100.0%
Total						
	White					
	n	2704 _a_	2765 _b_	3145 _c_	2122 _d_	10,736
	% of total	15.9%	16.3%	18.5%	12.5%	63.1%
	% of year grouping	67.3%	61.8%	64.7%	58.2%	63.1%
	Black/African American					
	n	1037 _a_	1175 _a_	919 _b_	560 _c_	3691
	% of total	6.1%	6.9%	5.4%	3.3%	21.7%
	% of year grouping	25.8%	26.3%	18.9%	15.4%	21.7%
	Asian					
	n	80 _a_	142 _b_	158 _b_	204 _c_	584
	% of total	0.5%	0.8%	0.9%	1.2%	3.4%
	% of year grouping	2.0%	3.2%	3.2%	5.6%	3.4%
	Other/Multiracial					
	n	173 _a_	351 _b_	621 _c_	746 _d_	1891
	% of total	1.0%	2.1%	3.7%	4.4%	11.1%
	% of year grouping	4.3%	7.8%	12.8%	20.5%	11.1%
	American Indian/Alaska Native					
	n	10 _a_	8 _a_	18 _a_	14 _a_	50
	% of total	0.1%	0.0%	0.1%	0.1%	0.3%
	% of year grouping	0.2%	0.2%	0.4%	0.4%	0.3%
	Unknown					
	n	15 _a_	32 _b_	3 _c_	0 _c_	50
	% of total	0.1%	0.2%	0.0%	0.0%	0.3%
	% of year grouping	0.4%	0.7%	0.1%	0.0%	0.3%
	Total					
	n	4019	4473	4864	3646	17,002
	% of total	23.6%	26.3%	28.6%	21.4%	100.0%
Completion Rate (Total Completers/Total Completers + Total Non-Completers)						
	White	38.44%	36.34%	30.83%	32.72%	34.59%
	Black/African American	21.90%	27.23%	28.40%	20.46%	24.96%
	Asian	54.10%	40.91%	49.58%	29.80%	41.27%
	Other/Multiracial	45.58%	36.00%	34.63%	26.20%	32.58%
	Native American/Alaska Native	42.86%	83.33%	53.33%	33.33%	50.00%
	Unknown	53.85%	44.44%	66.67%	100.00%	48.84%
	Total	34.71%	34.03%	31.58%	29.03%	32.43%

Note. Numbers with the same subscript letters are not statistically different from each other, and numbers with different subscript letters are statistically different at *p* < 0.05.

**Table 3:** Length of stay until treatment completion over time by racial group.

Race	Year Grouping	Mean (Days)	Std. Error
Asian	2004–2009	285.18	121.95
	2010–2014	201.31	101.11
	2015–2019	270.76	91.20
	2020–2024	550.93	104.43
Black/African American	2004–2009	405.37	51.50
	2010–2014	305.13	43.11
	2015–2019	310.91	48.22
	2020–2024	429.38	70.41
Native American/Alaska Native	2004–2009	504.66	404.47
	2010–2014	205.00	313.30
	2015–2019	384.11	233.52
	2020–2024	290.00	350.28
Other/Multiracial	2004–2009	297.79	85.58
	2010–2014	267.17	70.41
	2015–2019	342.37	53.73
	2020–2024	492.83	55.21
White	2004–2009	497.91	24.86
	2010–2014	452.01	25.9
	2015–2019	553.31	26.29
	2020–2024	960.56	31.20

Note. There was a significant difference between groups across time on length of stay (*F*(5, 4198) = 24.605, *p* < 0.011) and a significant interaction between racial group and year grouping (*F*(14, 4198) = 1,161,919.35, *p* = 0.003). White patients stayed in treatment significantly longer than Other/Multiracial, Asian, and Black/African American patients.

**Table 4:** Comparison of the racial distribution in the present sample between 2020 and 2024 and the current community representation in 2025.

Race	Racial Distribution of Substance Use Patients, 2020–2024	Current Racial Distribution of Individuals in New York State, 2025
White	58.2%	68.5%
Black/African American	15.4%	17.7%
American Indian/Alaska Native	0.4%	1.1%
Asian, Native Hawaiian, or Other Pacific Islander	5.6%	9.8%
Two or more races	20.5%	2.9%

## Data Availability

Due to the sensitive nature of these patient data, raw data that are de-identified are only available upon request from the corresponding author.

## Abbreviations

The following abbreviations are used in this manuscript:

SUD	Substance use disorder
AUD	Alcohol use disorder
